# Supplementary figures and images for: Force-dependent development of the myodural bridge in rats: The impact of Integrin α7
Source: PLoS One. 2025 Aug 4;20(8):e0329754. doi: 10.1371/journal.pone.0329754 (PMC12321098; doi:10.1371/journal.pone.0329754)

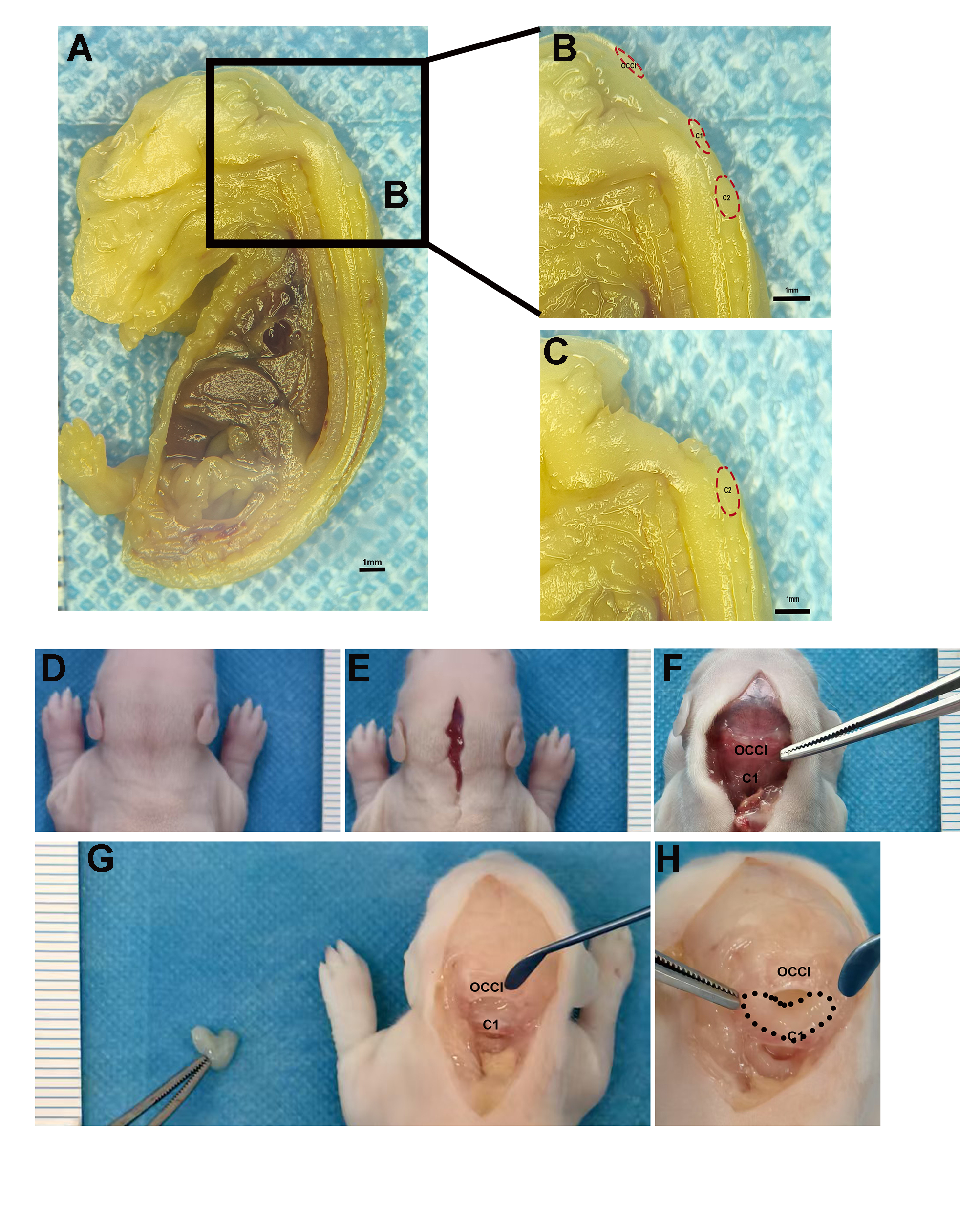

Supplement: S1 Fig — (A) Gross view of the E16 embryo tissue specimen, with the black box marking the area for locating key anatomical structures. (B) Magnified view of the boxed area in Figure A, for assisting in the accurate identification of MDBC – related structures at the embryonic stage. The dotted circles represent C1, C2, and OCCI, respectively. (C) The area from the inferior margin of the occipital bone to the atlas is the MDBC sampling site at the E16 stage. (D) Neck of a P7 neonatal rat before surgery. (E) Surgical incision made on the neck. (F) Superficial muscles dissected to expose the rectus capitis dorsalis major muscle, allowing clear visualization of the occipital bone and atlas. (G) The tissue in the atlanto – occipital space is dissected from the cephalic to the caudal direction. (H) Demonstration of placing the tissue back to its original position. OCCI: occipital bone. C1: atlas. C2:axis. (TIF) [file pone.0329754.s001.tif]

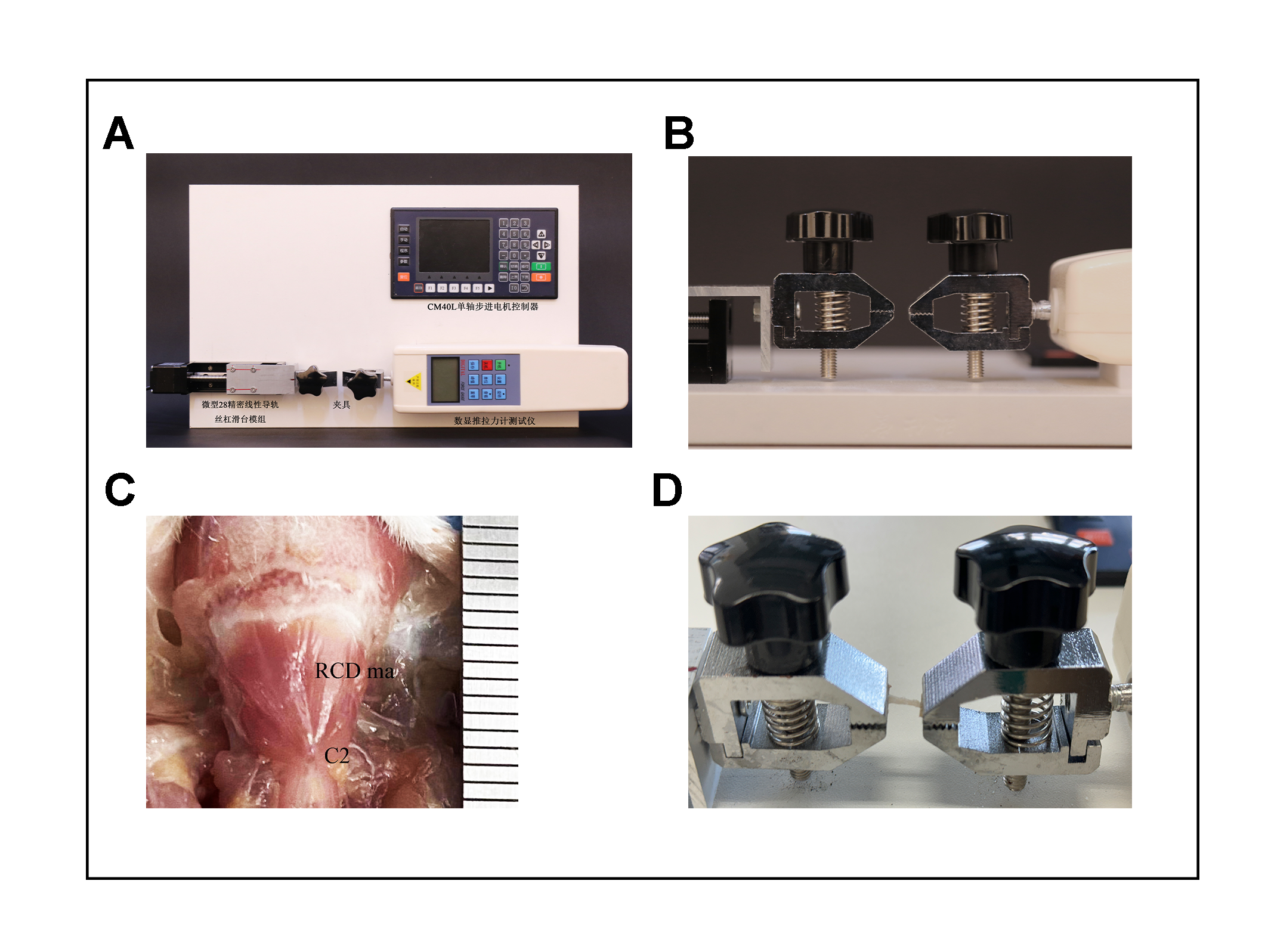

Supplement: S2 Fig — (A) Biomechanical Testing Device with 20 N capacity load cell. (B) Fixtures in Biomechanical Testing Device. (C) Exposed the RCDma and RCDmi. (D) Apply bandages to the cranial and caudal sides of the RCDmi and then place it in the fixture to perform uniaxial stretching. (TIF) [file pone.0329754.s002.tif]

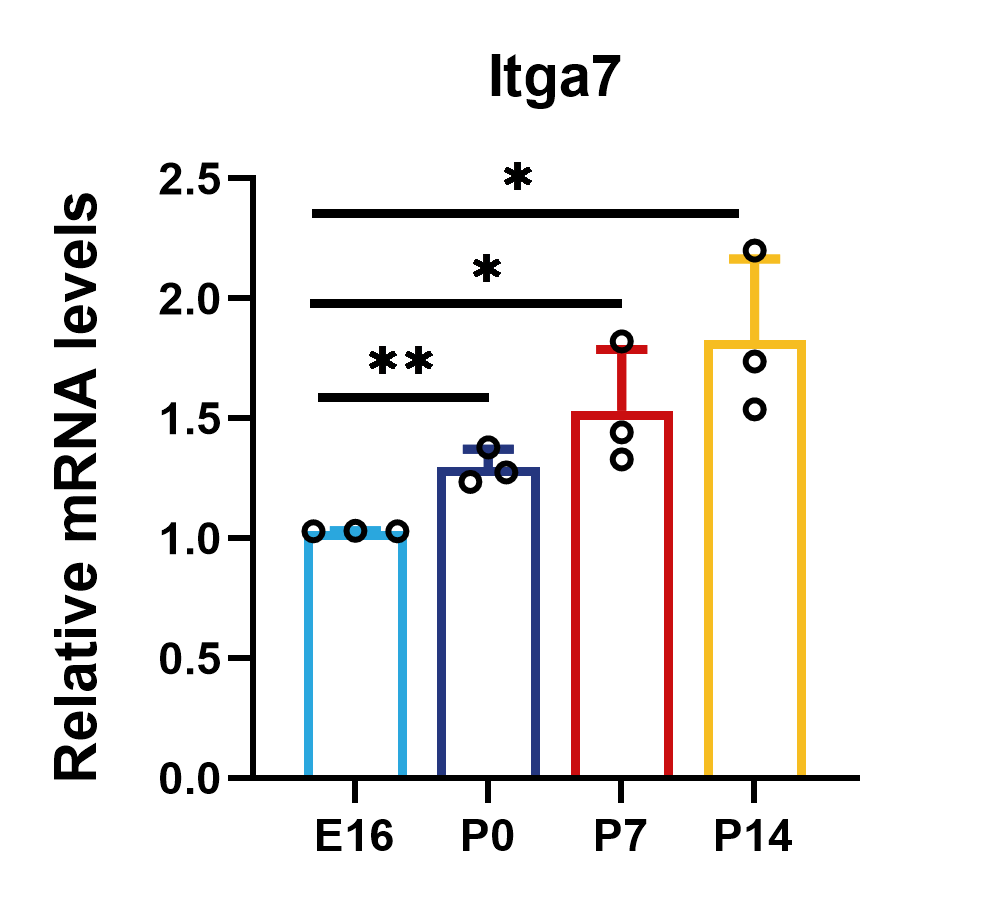

Supplement: S3 Fig — Itga7 expression in rats at four critical stages: embryonic day 16 (E16), postnatal day 0 (P0), postnatal day 7 (P7), and postnatal day 14 (P14) were evaluated by qRT-PCR. The expression of Itga7 increased progressively during MDBC development (*p < 0.05, **p < 0.01). (TIF) [file pone.0329754.s003.tif]

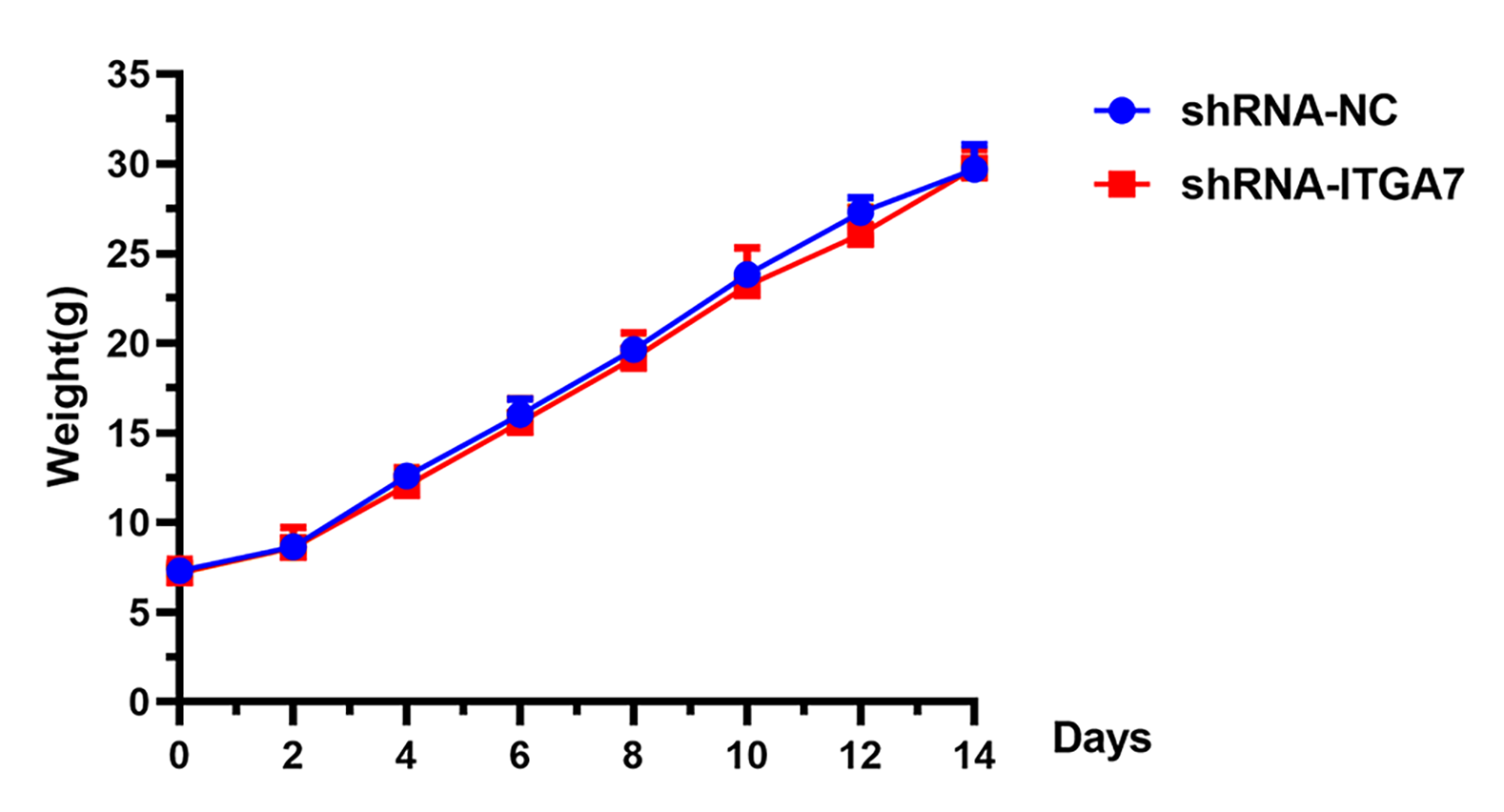

Supplement: S4 Fig — The body weights of the rats during the developmental process showed no significant difference between the two groups (p > 0.05). (TIF) [file pone.0329754.s004.tif]

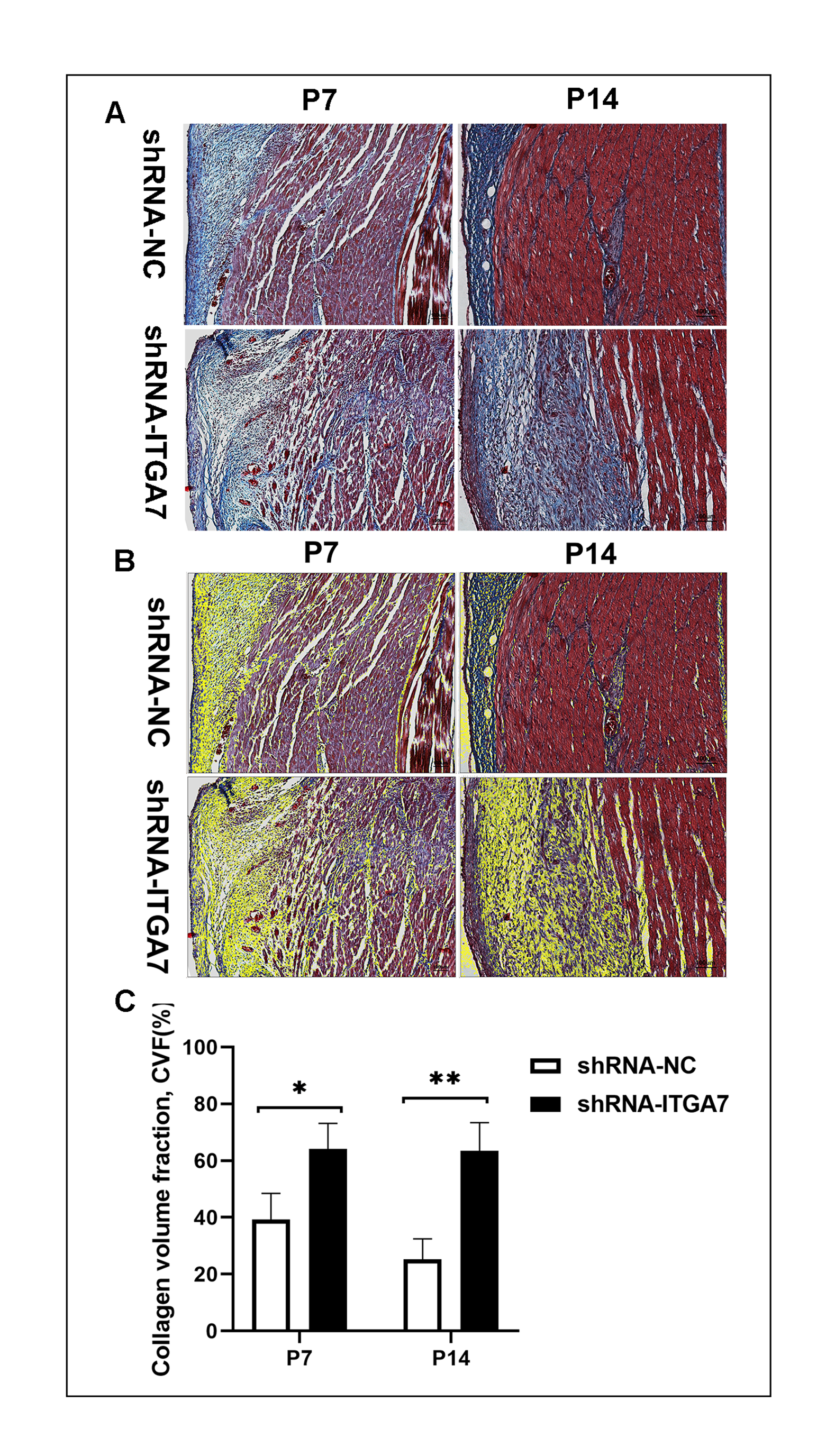

Supplement: S5 Fig — (A) Masson’s trichrome staining of the dorsal atlanto-occipital interspace in rats at P7 and P14 after localized ITGA7 knockdown. (B) The fibers of the dorsal atlanto-occipital interspace are circled in yellow by Image J software. The area of fibers are significantly increased in the shRNA-ITGA7 group. (C) Comparison of the area of the fibers between the shRNA-NC group and the shRNA-ITGA7 group. (*p < .05; ns: no statistical significance). (TIF) [file pone.0329754.s005.tif]

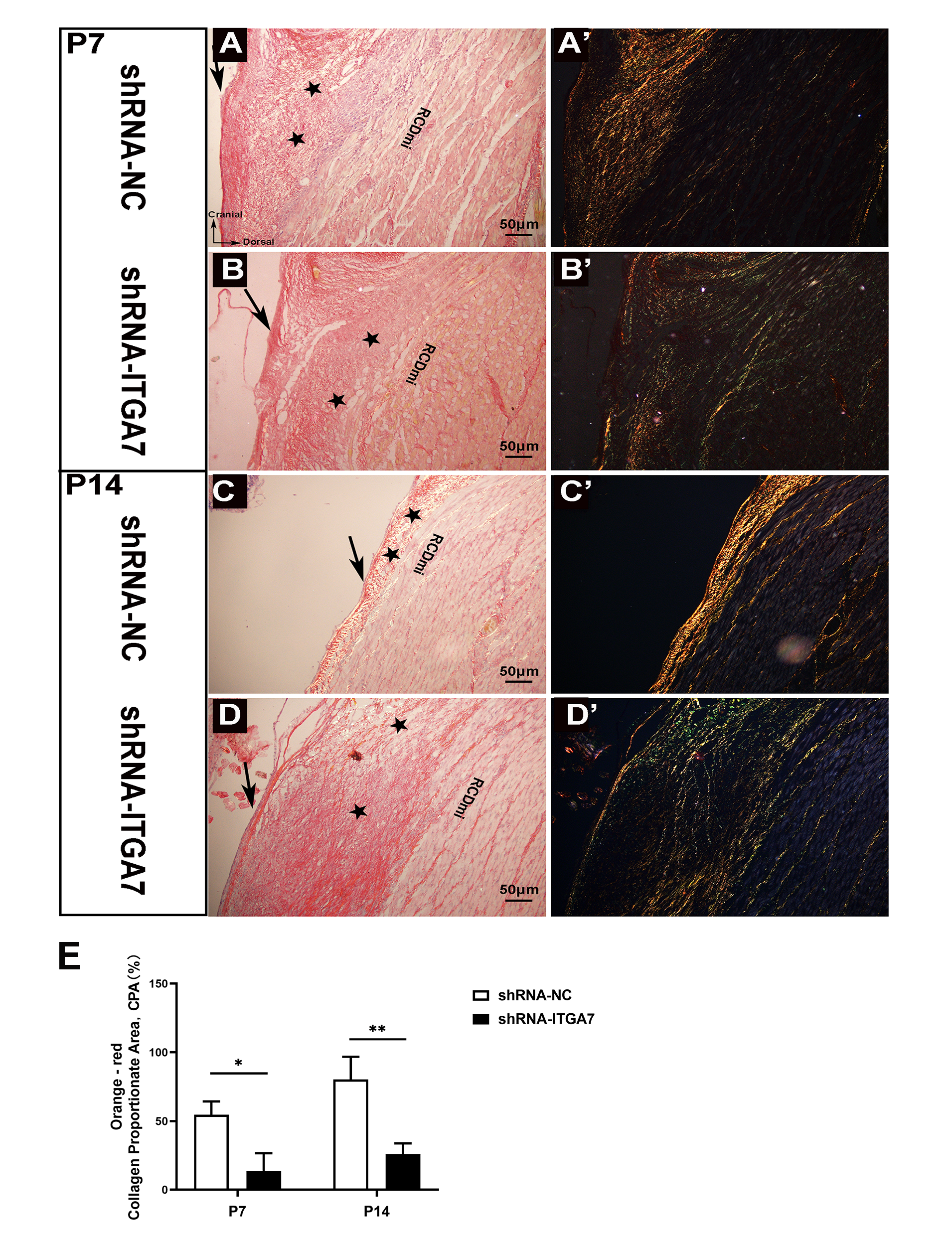

Supplement: S6 Fig — (A-D) were captured using a normal light microscope. (A’-D’) represent the same section observed under a polarized light microscope. (A and A’, B and B’) corresponded to the shRNA-NC and shRNA-ITGA7 group at P7, respectively. (C and C’, D and D’) corresponded to the shRNA-NC and shRNA-ITGA7 group at P14, respectively. (A’) MDB fibers in the shRNA-NC group presented birefringence ranging form yellow to red. (C’) The red birefringence of MDB fibers was intensified at P14. (B’ and D’) MDB fibers in the shRNA-ITGA7 group displayed a weak yellow birefringence, with an increase greenish collagen type III. (E) Proportion of orange – red fibers from Sirius Red staining between the shRNA - NC group and the shRNA - ITGA7 group. (* p < 0.05) All image acquisition parameters, including exposure time (150 ms) and light sensitivity (ISO 200) were consistent. RCDmi: rectus capitis dorsal minor muscle. ★: MDB fiber. ↑ : SDM. (TIF) [file pone.0329754.s006.tif]
