# Supplementary material for: Force-dependent development of the myodural bridge in rats: The impact of Integrin α7
Source: PLoS One. 2025 Aug 4;20(8):e0329754. doi: 10.1371/journal.pone.0329754 (PMC12321098; doi:10.1371/journal.pone.0329754)
Supplement: S1 Table — (DOCX) [file pone.0329754.s007.docx]

Table S1 The target sequences for stable knockdown of ITGA7.

Name Target sequences

ShRNA-1 5’-GCTAATGTGCAGAAGGAAAGC-3’

shRNA-2 5’-GCCACGAACAATTTGGGTTCT-3’

shRNA-3 5’-GCACCTCTGGAATCACCATTG-3’
