# Supplementary material for: Force-dependent development of the myodural bridge in rats: The impact of Integrin α7
Source: PLoS One. 2025 Aug 4;20(8):e0329754. doi: 10.1371/journal.pone.0329754 (PMC12321098; doi:10.1371/journal.pone.0329754)
Supplement: S2 Table — (DOCX) [file pone.0329754.s008.docx]

Table S2 Primer sequences were designed as follows

Gene Primer sequences

Col1a1 forward TGACGCATGGCCAAGAAGA

reverse CGTGCCATTGTGGCAGATAC

Col1a2 forward CATCGGTGGTACTAAC

reverse CTGGATCATATTGCACA

Lama2 forward GGGACACGAACGATGAGGAAA

reverse TTTTCACTTCGATGGGCTGCT

Tgfb1 forward CCTGGAAAGGGCTCAACAC

reverse CAGTTCTTCTCTGTGGAGCTGA

Scx forward AACACGGCCTTCACTGCGCTG

reverse CAGTAGCACGTTGCCCAGGTG

Pdgfra forward ACGTCTGGTCTTATGGCGTTCT

reverse CATCCTGTATCCGCTCTTGATCT

Gapdh forward CTACACTGAGGACCAGGTTGTCT

reverse GTTGCTGTAGCCATATTCATTGTC
