# Supplementary material for: Force-dependent development of the myodural bridge in rats: The impact of Integrin α7
Source: PLoS One. 2025 Aug 4;20(8):e0329754. doi: 10.1371/journal.pone.0329754 (PMC12321098; doi:10.1371/journal.pone.0329754)
Supplement: S3 Table — (DOCX) [file pone.0329754.s009.docx]

### Summary table of main quantitative results

| Measurement Index | ShRNA-NC  (Mean ±SD） | ShRNA-ITGA7  (Mean ±SD） | P-value |
| --- | --- | --- | --- |
| Collagen volume fraction (%)-P7 | 39.20±9.232 | 64.11±9.039 | 0.029 |
| Collagen volume fraction (%)-P14 | 25.21±7.287 | 63.44±9.932 | 0.006 |
| Orange-red Collagen Proportionate Area, CPA(%)-P7 | 54.62±9.636 | 13.64±12.955 | 0.012 |
| Orange-red Collagen Proportionate Area, CPA(%)-P14 | 80.24±16.530 | 25.98±7.795 | 0.007 |
| Tensile breaking force (N)-P7 | 0.19±0.006 | 0.12±0.031 | 0.024 |
| Tensile breaking force (N)-P14 | 0.35±0.133 | 0.33±0.047 | 0.818 |
| Col1a1 gene expression | 1.01±0.006 | 0.97±0.307 | 0.827 |
| Col1a2 gene expression | 1.00±0.004 | 0.66±0.232 | 0.025 |
| Lama2 gene expression | 1.01±0.015 | 0.75±0.151 | 0.041 |
| Tgfb1 gene expression | 1.03±0.053 | 0.71±0.146 | 0.007 |
| Scx gene expression | 1.01±0.014 | 0.96±0.259 | 0.680 |
| Pdgfra gene expression | 1.00±0.002 | 3.37±1.044 | 0.017 |
