# Supplementary material for: Force-dependent development of the myodural bridge in rats: The impact of Integrin α7
Source: PLoS One. 2025 Aug 4;20(8):e0329754. doi: 10.1371/journal.pone.0329754 (PMC12321098; doi:10.1371/journal.pone.0329754)
Supplement: Attachment — Submitted filename: Manuscript_v2-annotated.docx [file pone.0329754.s010.docx]

**Are extreme habitats an opportunity for speciation? First molecular and morphological evidence of an extremophile fish in a brackish** **rheocrene**

Sevan Ağdamar^1,*^, Gülşah Saç^2^, Deniz Anıl Odabaşı^3^, Müfit Özuluğ^2,4^

^1^ Department of Forestry, Bayramiç Vocational School, Çanakkale Onsekiz Mart University, Çanakkale, Türkiye

^2^ Department of Biology, Faculty of Science, İstanbul University, İstanbul, Türkiye

^3^ Department of Marine and Inland Water Sciences, Faculty of Marine Science and Technology, Çanakkale Onsekiz Mart University, Çanakkale, Türkiye

^4^ Center for Research and Practice in Natural Riches, İstanbul University, İstanbul, Türkiye

Correspondence: [sevanagdamar@comu.edu.tr](mailto:sevanagdamar@comu.edu.tr)

SA: 0000-0002-1268-0379

GS: 0000-0001-9988-1116

DAO: 0000-0003-0738-2606

MÖ: 0000-0002-1437-3890

**Abstract**

The small killifish *Aphanius almiriensis* Kottelat, Barbieri & Stoumboudi, 2007 inhabits from brackish springs to estuaries and coastal lagoons in the eastern Mediterranean basin. The present study focused on describing *Aphanius almiriensis* for the first time from a brackish rheocrene spring (Tuzla, Çanakkale, NW Türkiye) where exhibits extreme salinity (61.14 ppt in January, 2023) and temperature (42.7°C in July, 2023) conditions. The obtained specimens of killifish in the sampling site, with a slightly convex dorsal head profile and a more angular ventral head profile, is similar in this respect to specimens from Italy and Greece. In addition, the two scales on the in frontal area were not in contact with each other, but it was observed that these two scales were in contact with each other in only two individuals. Genetic analyses indicate that the species exhibits a minimum p-distance of 3.1% from *Aphanius fasciatus* in the mitochondrial cytochrome c oxidase subunit I (COI) barcode region. Both molecular and morphological characteristics of this newly described population are consistent with previously reported populations from Italy and Greece.

**Keywords:** Killifish, Species identification, COI, Restricted habitat, Extreme conditions

**Introduction**

Following molecular studies in recent years, the definition, limits and contents of the genus *Aphanius*, previously included in the family Cyprinodontidae, have been changed from a large genus to a much more restricted one [1]. The genus currently recognised in the family Aphaniidae, comprising eight monophyletic genera *Aphaniops*, *Aphanius*, *Anatolichthys*, *Apricaphanius*, *Esmaeilius*, *Kosswigichthys*, *Paraphanius*, and *Tellia*, is represented by only two species, *Aphanius fasciatus* and *Aphanius almiriensis*, distributed in the Mediterranean basin [2-4]. The Tethyan relict members of the genus inhabit the area with a rather surprising biogeographic pattern, preferring fresh and brackish or hypersaline water springs/coastal streams, lagoons, tidal channels, swamps, salt marshes and estuaries [2, 5].

Because species living in fresh-, brackish- or hypersaline- water bodies often exhibit narrow habitat specificity, they are of great interest for understanding the evolutionary mechanisms of differentiation [2]. Extreme water salinity or temperature conditions limit the presence of a variety of fish in water bodies and fish can only adapt to these extreme environments through phenotypic and physiological changes [6, 7]. Killifishes, comprising more than 1,200 species, are a group of fishes that are well adapted to these extreme conditions and are found worldwide in tropical and temperate, freshwater and estuarine habitats, from desert ecosystems to thermal springs and hypersaline waters [8-10].

Until recently, *Aphanius almiriensis* was considered under the species *Aphanius fasciatus*, which is morphologically very similar and is found in all coastal regions of the Mediterranean. However, *Aphanius almiriensis* was described as a new species from the Peloponnese (Greece) by Kottelat et al. (2007), immediately after Triantafyllidis et al. (2007) found two well-separated molecular groups in Greek *A. fasciatus* [4]. It has recently been recorded from a small coastal cenote in Italy, outside its known range, as well [4]. According to Triantafyllidis et al. (2007), *A. almiriensis* is more common in the Aegean basin and it is possible that it can also be identified on the Turkish coast.

To date, there has been no scientific study of the killifish population, which is known to inhabit the Tuzla brackish rheocrene spring area and is believed to be the species *A. fasciatus.* The objectives of the present study are i) to identify the *Aphanius* population living in the Tuzla rheocrene spring on a species basis using both morphological and molecular analyses and ii) to reveal the extreme conditions that are effective in exhibiting narrow habitat specificity.

**Materials and Methods**

***Study area and sampling***

Çanakkale–Tuzla geothermal field, situated approximately 80 km southwest of Çanakkale and 5 km inland from the Aegean coast, is characterized as a volcanic region [11, 12]. The thermal waters, with a surface discharge temperature of 34 and 80°C, originate from two distinct reservoirs: a shallow volcanic reservoir located at a depth of 330–350 meters, and a deeper granitic reservoir at approximately 1020 meters. The thermal waters of Tuzla are distinguished by their exceptionally high dissolved salt concentration, reaching up to 68 g/L, and their enriched content of calcium (Ca), sodium (Na), potassium (K), strontium (Sr), and chloride (Cl), surpassing those of other thermal water regions in Western Anatolia [11, 13-16].

Tuzla village located in the southwest of the Biga Peninsula (Çanakkale, Türkiye) is a region of thermal water outlets. On the tectonic fault line at the foot of a mountain near to village, geothermal evaporation causes the spring water to reach a temperature of around 70°C. Two independent springs have been observed in the region, and they are characterised as rheocrene waters, which are formed by a combination of hot and cold-water sources. These springs, which flow separately through narrow channels, can reach the Tuzla Stream, which flows into the northern Aegean Sea. Although the springs flow throughout the year, their volume is reduced by evaporation during the summer months. The study was carried out in these two rheocrene springs (Figure 1, 2):

*Site 1* (39.569036N 26.172661E) is in the Tuzla village. It is a shallow (10-30 cm depth) rheocrene spring flowing through a narrow canal (30-40 cm width). This rheocrene spring is fed both by weak water at a temperature of about 70°C, which is discharged by geothermal evaporation, and by cold leakage water in the rock. The site is surrounded by a thermal bath and a goat farm.

*Site 2* (39.576950N 26.169161E) is located 1 km north of the Tuzla village on the road. This rheocrene spring is also fed both by weak water at a temperature of about 70°C and by cold leakage water in the rock. The first place where these waters flowing through the rocks mix is a small puddle, 1 m in diameter and about 30 cm deep, which spreads out and becomes shallow, forming a marshy area (about 20 m in diameter).

The field surveys of this study were conducted within the scope of the project titled "Species Identification of *Aphanius sp.* Inhabiting Extreme Conditions in the Thermal Spring of Tuzla Village (Çanakkale, Türkiye) using Morphological and Molecular Methods" funded by the Research Fund of Çanakkale Onsekiz Mart University (Project No: FBA-2022-4179). Accordingly, the treatment of collected fish specimens was consistent with the Republic of Türkiye’s animal welfare laws and guidelines, and the experiments conducted with the permission of Çanakkale Onsekiz Mart University Committee of Animal Experiments Local Ethics (Decision ID: 2022/05-03).

The study area was visited seasonally between January and October 2023, and fish samples were collected using a scoop net with a mesh size of 3 mm. The collected specimens were sacrificed with an overdose of clove oil. For the morphological analyses, some specimens were fixed in 4% formaldehyde solution and then stored in 70% ethanol. The rest were fixed directly in 99% ethanol for molecular analysis. The physico-chemical parameters of the springs were measured using a multi-parameter (Eutech CyberScan 600).

***Morphological analyses***

Kottelat & Freyhof (2007) were followed for measurements and counts. All measurements were made with a digital caliper point-to-point and recorded to the nearest 0.1 mm. Standard length (SL) was measured from the tip of the snout to the end of the hypural complex. The length of the caudal peduncle was measured from behind the base of the posterior anal-fin ray to the end of the hypural complex, at mid-height of the caudal-fin base. Lateral line scales were counted from the anteriormost scale (the first one to touch the shoulder girdle) to the last scale at the end of the hypural complex. Scales on the caudal fin itself are indicated by “+”. For the comparisons, morphological data for *A. almiriensis* inhabits Greece and Italy are based on Kottelat et al. (2007) and Valdesalici et al. (2019).

***DNA extraction, PCR, and sequencing***

Genomic DNA was extracted from caudal fin tissue using Qiagen DNeasy Blood & Tissue Kit. Species-specific primer pairs (COI-F: 5'-TCAACCAACCACAAAGACATTGGCAC-3' and COI-R: 5'-TAGACTTCTGGGTGGCCAAAGAATCA-3') were designed to amplify only the DNA of target species from tissue content. The specificity of primer pairs was tested in silico using the ecoPCR [17] and primerBLAST [18] software. DNA amplifications were performed in 25 µl volumes, each containing: 2.5 µl of 10X Taq Buffer with KCl (100 mM Tris-HCl, 500 mM KCl, pH 8.8), 2.5 µl of MgCl2 (25 mM), 0.5 µl of dNTPs (10 mM), 0.5 µl of each primer (10 pM/µl), 1 U of Taq polymerase (5 U/µl), and 1 µl of DNA (50 ng/µl). PCR amplifications were performed in Eppendorf Mastercycler® with the following cycling conditions [19]: preliminary denaturation at 95°C for 5 min followed by 35 cycles consisting of denaturation at 95°C for 1 min, primer annealing at 54°C for 1 min, primer extension at 72°C for 1 min and final extension step at 72°C for 10 min. Sanger sequencing was performed by BM Labosis Ltd. Co. (Ankara, Turkey) with reverse primer (COI-R).

***Molecular data analyses***

Nucleotide sequences were aligned using the ClustalW algorithm in MEGA X [20]. DNA sequence data were submitted to GenBank and the accession numbers PP218677-PP218686 were assigned. A molecular taxonomic cladogram was generated in MEGA X [20] maximum likelihood (ML) algorithm using a HKY substitution model with 1000 bootstrap replications. Genetic distances between species were calculated in MEGA X [20] using the p-distance model with 1000 bootstrap replications. Reference sequences of the species on cladogram were retrieved from GenBank (Table 1).

**Results**

***Material examined***

IUSHM 2024-1471, 14 ind., 27-32 mm SL; Türkiye, Çanakkale prov., Tuzla thermal spring, 1 km north of Tuzla village on the road; April 2023 – IUSHM 2024-1472, 17 ind., 24-28 mm SL; Türkiye, Çanakkale prov., Tuzla thermal spring 1 km north of Tuzla village on the road; January 2023 – IUSHM 2024-1473, 14 ind., 19-40 mm SL; Türkiye, Çanakkale prov., Tuzla thermal spring 1 km north of Tuzla village on the road; July 2023 – IUSHM 2024-1474, 10 ind., 24-32 mm SL; Türkiye, Çanakkale prov., Tuzla thermal spring 1 km north of Tuzla village on the road; August 1993 – IUSHM 2024-1477, 18 ind., 14-26 mm SL; Türkiye, Çanakkale prov., Tuzla thermal spring near to thermal bath in Tuzla village; January 2023

***Additional material***

IUSHM 2024-1475, 12 ind., 35-48 mm SL; Türkiye, İstanbul prov., Küçükçekmece Lagoon; June 1973 – IUSHM 2024-1476, 2 ind., 54-63 mm SL; Türkiye, İstanbul prov., Büyükçekmece Lagoon; June 1981

***Description***

See Figure 3-6 for general appearance of the species and Table 2 for morphometric data of *A. almiriensis*. Relatively large looking head. Lower jaw slightly curved upwards. Teeth tricuspid, median tip longer than laterals. Dorsal profile slightly convex from nape to dorsal‐fin origin, ventral profile more convex than dorsal profile. The deepest point of the body is located between the pectoral and pelvic fins. Pectoral fin rounded, its posterior margin reaching pelvic‐fin origin. Pelvic fin not reaching anal‐fin origin. Caudal peduncle compressed laterally. Anal fin origin below vertical of 3^rd^ or 4^th^ branched dorsal‐fin ray in male 3^rd^ or 4^th^ in female. Dorsal and anal fins rounded, not reaching caudal‐fin base. Caudal fin rounded or sub‐truncate. Dorsal and anal fins with 7½(1), 8½(9), 9½(5) branched rays. Caudal fin with 7 + 7(1), 7 + 8(2), 8 + 7(3), 8 + 8(9) branched rays. Pectoral fin with 14(10), 15(5) and pelvic fin with 6(2)–7(13) rays. Trunk and head covered by large and cycloid scales. Flank with 24 (2), 26 (11), 27 (2) scales along lateral series. Two or three additional rows of small scales on anterior caudal‐fin base. 3-4 scales rows between dorsal‐fin and lateral series scales, 5-6 lateral series scales and pelvic-fin. Caudal peduncle with 15(1)–16(14) circumpeduncular scales. Peritoneum black. Cephalic neuromasts in interorbital area in a deep open groove (Figure 7).

*Preserved Male colour*: Upper part of head brownish. Lower part of head and pectoral fin base silvery. Lower cheek, breast and belly whitish. Dorsal portion of head and flank brown. Flank with 9–12 brown to dark‐grey bars. First bar at vertical of pectoral‐fin base, last bar shortly anterior to caudal‐fin origin. Pectoral and pelvic fins hyaline. Anal fin hyaline. Dorsal‐fin hyaline, black pigments on the branched rays. A black dorsal‐fin margin, wide anteriorly, narrow or absent at last dorsal‐fin rays. Caudal fin pale hyaline, a dark vertical band in some samples.

*Female colour*: Upper part of head brownish grey. Lower part of head and pectoral fin base whitish silvery. One series of 7–11 dots along lateral mid‐ line, vertically elongated in some individuals. One dot at middle of caudal‐fin base in most individuals. Two elongated small confused black dots at base of caudal fin in some individuals. All fins hyaline.

***Molecular data***

Figure 8 illustrates a molecular cladogram reconstructed from mitochondrial cytochrome c oxidase subunit I (COI) gene sequences of 10 *A. almeriensis* specimens and their closest congeneric species, including sequences retrieved from GenBank. The analysis confirmed the monophyly of all *A. almeriensis* specimens with robust statistical support (e.g., high bootstrap values). The DNA barcodes of *A. almiriensis* share the same nucleotide sequence and cluster together (Figure 8), showing a 3.1% genetic divergence from their closest relative, *A. fasciatus*, as indicated in Table 5. Notably, *Aphanius almeriensis* populations sampled in Çanakkale (Türkiye) formed a genetically distinct clade, clearly differentiated from other killifish species represented in the GenBank dataset.

The molecular cladogram, reconstructed through maximum likelihood (ML) analysis of COI sequences and supported by high bootstrap values, confirmed that molecular identification using COI data aligns with traditional taxonomic classifications for this putative species. Interspecific genetic divergence values among these congeners were consistent with established thresholds for freshwater fish species. Ten COI sequences obtained from *Aphanius* specimens exhibited high intraspecific similarity, forming a cohesive cluster distinct from their closest taxonomic relatives within the genus. The observed genetic distances between these clusters align with values characteristic of evolutionarily independent species (Table 3).

***Habitat notes***

These springs, which are the mixture of hot and cold water flowing through the rocks, is very shallow and has a relatively low flow compared to any stream (Figure 2). Salinity and temperature values of these rheocrene springs were measured at high levels with 61.14 ppt in January and 42.7°C in July (Table 4). It is important to note that the salinity and temperature values obtained are higher than those of seawater (approximately 38‰ in the Mediterranean Sea), illustrating that the fish have adapted to extreme conditions. The absence of *A. almiriensis* in samplings from the Tuzla Stream suggests no potential hybridization with its marine conspecific population (i.e., *A. fasciatus*), and indicates that *A. almiriensis* is entirely isolated.

**Discussion**

All species in Aphaniidae are morphologically homogeneous and have similar scale patterns, fin positions, and meristic characters [3]. There are limited literatures on *Aphanius almiriensis* and the species was first described from an open water spring at Almiri beach in Peloponnese and the Meligou lagoon from Greece [28]. According to Kottelat et al. (2007), *A. almiriensis* is morphologically very similar to *A. fasciatus*, but differs from it with a stouter caudal peduncle, wider interorbital distance and larger eyes.

They have also observed differences between the two species in the number and organisation of neuromasts in the interorbital areas and frontal scales. *Aphanius almiriensis* has 2 neuromasts in a deep groove on each side in the interorbital area and the 2 most anterior scales on the frontal area do not touch each other. In contrast, in *A. fasciatus* there are 3 neuromasts in 3 pores, and the 2 most anterior scales on the frontal area touch each other. However, only one *A. almiriensis* individual had a partially closed groove with 2 neuromasts inside [28]. In another study, the first record of *A. almiriensis* was given from Italy [4] and that population was recognised with 2 rostral neuromasts, 2-4 supraorbital neuromasts in a deep groove, and three postorbital neuromasts in a rounded, deep, open groove, whereas *A. fasciatus* has 2+4 rostral neuromasts, 3 neuromast in 3 supraorbital pores and 2-4 neuromasts in a curved groove [4]. In this study, it was observed that the number and structure of the supraorbital neuromasts of the Tuzla populations were compatible with Italy and Greece *A. almiriensis* (2-3 supraorbital neuromasts in a deep groove Tuzla *A. almiriensis*). But postorbital neuromasts is 3-4 and they are in a curved (ovoid in few samples) open shallow groove. Only two specimens have 2 postorbital neuromasts and one in a round groove while the other in a channel. The Tuzla specimens therefore differ from the *A. almiriensis* in Greece and Italy in the number and groove shape of the postorbital neuromasts (vs. 1-2 in round groove in Greece population and 3 in Italy population). In the samples obtained from Tuzla in 1993, neuromasts were similar to the current samples. After morphological investigating the Küçükçekmece and Büyükçekmece specimens previously identified as *Aphanius fasciatus* [29, 30], it was found that these specimens are also *A. almiriensis* and their neuromasts are similar in number and shape to the Tuzla population. Both lagoon specimens are expressly larger than the Tuzla specimens. Two individuals (one male and one female) from the Büyükçekmece Lagoon have two supraorbital neuromasts, but the deep groove is partially closed.

*Aphanius almiriensis* in the Tuzla, with a slightly convex dorsal head profile and a more angular ventral head profile, is similar in this respect to specimens from Italy and Greece. In addition, the two scales on the in frontal area were not in contact with each other, but it was observed that these two scales were in contact with each other in only two individuals. Especially in the larger Tuzla and Büyükçekmece-Küçükçekmece specimens, the cephalic scales were very well developed and extended to the edge of the supraorbital and postorbital neuromasts.

Metric analysis of the Tuzla specimens revealed that the distance between the ventral and anal fins differed between males and females. The beginning of the anal fin is more anterior in males; hence the length of the caudal peduncle is greater in males. These results are also in agreement with Kottelat et al. (2007).

According to Kottelat et al. (2007) and Valdesalici et al. (2019), males of *A. almiriensis* do not have a vertical band on the caudal fin. Only two individuals (>24mm) caught in Tuzla in July (2023) showed a band on the caudal fin, which was very faint and less visible in the fixed specimens. However, almost all male individuals (>35 mm) from Büyükçekmece and Küçükçekmece lagoons showed a vertical band on the caudal fin. These results indicate that the vertical band on the caudal fins of male individuals occurs only in larger individuals. In the females, 7-11 large spots on the lateral midline are weakly elongated vertically in some individuals. Behind these spots there is an irregular, faint, dark mid-lateral inner stripe.

The maximum likelihood analysis of the COI barcode data yielded consistent cladogram with strong bootstrap support. In the molecular identification of this potential species through DNA barcoding, it has been shown that these markers effectively delineate species in accordance with traditional taxonomic classifications. The genetic distance observed among these congeners falls within the ranges reported for freshwater fish [31]. DNA barcodes derived from *A. almiriensis* specimens are identical and form a distinct cluster, separate from those of the nearest *Aphanius* species. The genetic distance values indicate that *A. almiriensis* is likely an independent species, as the likelihood of conspecificity significantly decreases with a divergence of 2% in fish [32].

**Conclusion**

Considering all these data, this study documents the first molecular and morphological characteristics of *A. almiriensis* from Turkish waters. Although it is widely distributed in the Mediterranean basin from Italy to Türkiye, the water bodies where it has been identified are very limited. The species was assessed as being Least Concern (LC) following the IUCN criteria for the Red List. However, it is noted that current population trends are declining in the face of threats such as droughts, invasive non-native/alien species, diseases, pollution and natural system modifications (IUCN, 2025). The cyprinodontid fish *Aphanius almiriensis* confronts heightened extinction risks attributable to its narrowly constrained habitat and critically low abundance, both of which jeopardize the maintenance of genetic diversity. Unpublished monitoring data from Site 2 highlight a disproportionately female-skewed demographic structure within this population, a condition likely to intensify inbreeding depression and drive the population toward a genetic bottleneck. These findings underscore the urgency of adopting dual conservation priorities: mitigating habitat degradation while implementing genomic rescue strategies to bolster adaptive potential, thereby securing the population's evolutionary resilience. It is expected that both molecular and morphological studies in the basin, especially in water bodies such as lagoons and thermal/salty springs with extreme habitat conditions, will increase the population number of the species. In addition, further studies should focus on the genetic mechanisms of the species for these extreme conditions.

**References**

1. Kottelat M. & Freyhof J. Handbook of European freshwater fishes (p. 646). Cornol and Freyhof, Berlin, Publications Kottelat (2007).
2. Triantafyllidis A., Leonardos I., Bista I., Kyriazis I.D., Stoumboudi M. TH., Kappas I., Amat F. & Abatzopoulos T. J. Phylogeography and genetic structure of the Mediterranean killifish *Aphanius fasciatus* (Cyprinodontidae). *Mar. Biol.* **152**, 1159–1167 (2007).
3. Freyhof, J. & Yoğurtçuoğlu, B. A proposal for a new generic structure of the killifish family Aphaniidae, with the description of *Aphaniops teimorii* (Teleostei: Cyprinodontiformes). *Zootaxa*, **4810**, 421-451; <https://doi.org/10.11646/zootaxa.4810.3.2> (2020).
4. Valdesalici S., Brahimi A. & Freyhof J. First record of *Aphanius almiriensis* from Italy and notes on the distribution of *Aphanius fasciatus* (Teleostei: Aphaniidae). *J. Appl. Ichthyol.* **35**, 541-550; <https://doi.org/10.1111/jai.13873> (2019).
5. Hrbek, T. & Meyer, A. Closing of the Tethys Sea and the phylogeny of Eurasian killifishes (Cyprinodontiformes: Cyprinodontidae). *J. Evol. Biol.* **16**, 17–36; <https://doi.org/10.1046/j.1420-9101.2003.00475.x> (2003).
6. Eaton J. G., McCormick J. H., Stefan H. G. & Hondzo M. (1995). Extreme value analysis of a fish/temperature field database. *Ecol. Eng.* **4**, 289-305 (1995).
7. Wang Y. & Guo B. Adaption to extreme environments: a perspective from fish genomics. *Rev. Fish Biol. Fish.* **29**, 735-747;  [https://doi.org/10.1007/s11160-019-09577-9](%20https://doi.org/10.1007/s11160-019-09577-9) (2019).
8. Akbarzadeh, A., Mosayebi, A. N., Noori, A., Parto, P., Asadi, M., Yousefzadi, M., Dehghani, H. & Kamrani, E. Responses of the killifish (*Aphanius dispar*) to long-term exposure to elevated temperatures: growth, survival and microstructure of gill and heart tissues. *Mar. Freshw. Behav. Physiol.* **47**, 429-434 (2014).
9. Chiozzi, G., Stiassny, M. L. J., Alter, S. E., De Marchi, G., Mebrahtu, Y., Tessema, M., Asmamaw, B., Fasola, M., Bellati, A. (2018). Fishes in the desert: mitochondrial variation and phylogeography of Danakilia (Actinopterygii: Cichlidae) and *Aphanius* (Actinopterygii: Cyprinodontidae) in the Danakil Depression of northeastern Africa. *Mitochondrial DNA A*, **29**, 1025-1040; <https://doi.org/10.1080/24701394.2017.1404043> (2018).
10. Piller, K. R., Parker, E., Lemmon, A. R., Lemmon, E. M. Investigating the utility of Anchored Hybrid Enrichment data to investigate the relationships among the Killifishes (Actinopterygii: Cyprinodontiformes), a globally distributed group of fishes. *Mol. Phylogenetics Evol.* **173**, 107482; <https://doi.org/10.1016/j.ympev.2022.107482> (2022).
11. Baba, A., Yuce, G., Deniz, O. & Ugurluoglu, D. Y. Hydrochemical and Isotopic Composition of Tuzla Geothermal Field (Canakkale-Turkey) and its Environmental Impacts. *Environ. Forensics.* **10**, 144–161 (2009).
12. Erenoglu, R. C., Arslan, N., Erenoglu, O., & Arslan, E. Application of spectral analysis to determine geothermal anomalies in the Tuzla region, NW Turkey. *Arab. J. Geosci.* **12**, 1-15; <https://doi.org/10.1007/s12517-019-4599-2> (2019).
13. Baba, A. Geothermal environmental impact assessment with special reference to the Tuzla, Geothermal Area, Canakkale Turkey. Iceland: Geothermal Training Programme Book, pp. 75–114 (2003).
14. Karamanderesi, I. H. Hydrothermal alteration in well Tuzla T-2, Canakkale, Turkey, No. 3 (1986).
15. Şimsek, S. Geothermal potential in northwestern Turkey: Active tectonics of Northwestern Anatolia, The Marmata Poly-Project (1997).
16. Vengosh, A., Helvacı, C. & Karamanderesi, İ. H. Geochemical constraints for the origin of thermal waters from western Turkey. *Appl. Geochem.* **17**, 163-183 (2002).
17. Dejean T., Valentini A., Duparc A., Pellier-Cuit S., Pompanon F., Taberlet P. & Miaud C. Persistence of environmental DNA in freshwater ecosystems. *PLOS One,* **6**, e23398; <https://doi.org/10.1371/journal.pone.0023398> (2011).
18. Jerde C., Mahon A., Chadderton W. & Lodge D. Sight-unseen detection of rare aquatic species using environmental DNA. *Conserv. Lett*. **4**, 150-157; <https://doi.org/10.1111/j.1755-263X.2010.00158.x> (2011).
19. Keskin, E. & Atar, H.H. Molecular identification of fish species from surimibased products labeled as Alaskan pollock. *J. Appl. Ichthyol.* **28**, 811-814 (2012).
20. Kumar, S., Stecher, G., Li, M., Knyaz, C. & Tamura, K. MEGA X: Molecular evolutionary genetics analysis across computing platforms. *Mol. Biol. Evol.* **35**, 1547-1549; <https://doi.org/10.1093/molbev/msy096> (2018).
21. Geiger M. F., Herder F., Monaghan M. T., Almada V., Barbieri R., Barich, M., Berrebi P., Bohlen J., Casal-Lopez M., Delmastro G. B. *et al.* Spatial heterogeneity in the Mediterranean Biodiversity Hotspot affects barcoding accuracy of its freshwater fishes. *Mol. Ecol. Resour.* **14**, 1210–1221; <https://doi.org/10.1111/1755-0998.12257> (2014).
22. Landi M., Dimech M., Arculeo M., Biondo G., Martins R., Carneiro M., Carvalho G. R., Lo Brutto S. & Costa F. O. DNA barcoding for species assignment: the case of Mediterranean marine fishes. *PLOS One* **9**, e106135; <https://doi.org/10.1371/journal.pone.0106135> (2014).
23. Pfleiderer S. J., Geiger M. F. & Herder F. *Aphanius marassantensis*, a new toothcarp from the Kızılırmak drainage in northern Anatolia (Cyprinodontiformes: Cyprinodontidae). *Zootaxa* **3887**, 569–582; <https://doi.org/10.11646/zootaxa.3887.5.4> (2014).
24. Zarei, F., Masoumi, A. H., Al Jufaili, S. M. & Esmaeili, H. R. Contribution to the diversity and distribution of Aphaniops (Teleostei: Aphaniidae) in Oman freshwater ecoregions: units for taxonomy and conservation. *Biologia*, **78**, 851-863; <https://doi.org/10.1007/s11756-022-01289-z> (2023).
25. Teimori A., Esmaeili H.R., Zarei F. & Reichenbacher B. COI gene sequences confirm the taxonomic validity of the tooth-carp *Aphaniops hormuzensis* (Teleostei: Aphaniidae) from southern Iran. *Zool. Middle East* **68**, 34-40; <https://doi.org/10.1080/09397140.2021.1965071> (2022).
26. Freyhof J., Kärst H. & Geiger M. *Valencia robertae*, a new killifish from southern Greece (Cyprinodontiformes: Valenciidae). *Ichthyol. Explor. Freshw.* **24**, 289-298; (2014).
27. Vogiatzi E., Kalogianni E., Zimmerman B., Giakoumi S., Barbieri R., Paschou P., Magoulas A., Tsaparis D., Poulakakis N. & Tsigenopoulos C.S. Phylogeography of the Corfu killifish. *Biol. J. Linn. Soc.* **111**, 334-349; (2014).
28. Kottelat M., Barbieri R. & Stoumboud, M. Th. *Aphanius almiriensis*, a new species of toothcarp from Greece (Teleostei: Cyprinodontidae). *Rev. Suisse Zool.* **114**, 13-31 (2007).
29. Meriç, N. Fishes encountered in Büyükçekmece Lake, İstanbul. *İstanbul Üniv. Fen Fak. Mec.* **51**, 41-46 (1986a)
30. Meriç, N. Fishes encountered in Küçükçekmece Lake, İstanbul. *İstanbul Üniv. Fen Fak. Mec.* **51**, 33-39 (1986b).
31. Hubert, N., Hanner, R., Holm, E., Mandrak, N.E., Taylor, E., Burridge, M., Watkinson, D., Dumont, P., Curry, A., Bentzen, P. Identifying Canadian freshwater fishes through DNA barcodes. *PLoS ONE*, **3**, e2490; <https://doi.org/10.1371/journal.pone.0002490> (2008).
32. Ward, R. D. DNA barcode divergence among species and genera of birds and fishes. *Mol. Ecol. Res.* **9**, 1077-1085; <https://doi.org/10.1111/j.1755-0998.2009.02541.x> (2009).
33. IUCN 2025. The IUCN Red List of Threatened Species. Version 2024-2; <https://www.iucnredlist.org> (2025).

**Acknowledgements**

This study was supported by the Research Fund of Çanakkale Onsekiz Mart University (Project No: FBA-2022-4179). This study was also conducted with the permission of Çanakkale Onsekiz Mart University Committee of Animal Experiments Local Ethics (Decision ID: 2022/05-03). The authors thank Dr. Oya Özuluğ, Harun İnci and Özgün Deniz Yürekli for their kind helps during the field surveys.

Authors' contributions

SA: Substantial contribution in the concept and design of the study; contribution to field data collection; contribution to molecular and data analyses; contribution to manuscript preparation. GS: Contribution to field data collection and manuscript preparation. DAO: Contribution to field data collection and manuscript preparation. MÖ: Contribution to field data collection; contribution to data analyses and manuscript preparation. All authors have read and agreed to the recent version of the manuscript.

Data availability statement

The raw data supporting the conclusions of this article will be made available by the corresponding author on request.

Competing interests

The authors declare no conflicts of interest.

**Figure legends**

Figure 1. Map of the study area (Tuzla brackish rheocrenes, NW Türkiye) and sampling sites.

Figure 2. Images of the study area and sampling sites (A: Site 1, B: Site 2).

Figure 3. *Aphanius almiriensis,* IUSHM 2024-1473, (in live) 25 mm SL male (top), 24 mm SL female (bottom) from Tuzla rheocrene spring (Çanakkale, Türkiye).

Figure 4. *Aphanius almiriensis,* IUSHM 2024-1473, from the top, 25 mm SL, 23 mm SL, 18 mm SL, 18 mm SL male, from Tuzla rheocrene spring (Çanakkale, Türkiye).

Figure 5. *Aphanius almiriensis,* IUSHM 2024-1473, females, 30 mm SL (top), 27 mm SL (bottom), from Tuzla rheocrene spring (Çanakkale, Türkiye).

Figure 6. *Aphanius almiriensis,* IUSHM 2024-1475, 38 mm SL male Küçükçekmece Lagoon.

Figure 7. Two scales anterior frontal and supraorbital grove with two neuromast (top), postorbital curved grove with three (bottom). Please improve the English, the caption is not easily understandable.

Figure 8. Molecular cladogram based on the maximum likelihood (ML) analysis of pairwise genetic p-distances. Bootstrap values for 1000 replicates are shown above branches on the cladogram. Scale: 0.5% p-distance. Mark in bold the specimens of your study or add that the specimens of your study have GenBank codes starting with PP

**Table 1.** List of COI GenBank sequences of *Aphanius* species used in the molecular analysis of *A. almiriensis*

| **Species** | **Location** | **GenBank Acc. No.** | **Reference** |
| --- | --- | --- | --- |
| *Aphanius almiriensis* | Greece | KJ552360 | [21] |
| *Aphanius almiriensis* | Greece | KJ552640 | [21] |
| *Aphanius almiriensis* | Greece | KJ552525 | [21] |
| *Aphanius almiriensis* | Italy | MH410025 | Unpublished |
| *Aphanius almiriensis* | Italy | MH410027 | Unpublished |
| *Aphanius fasciatus* | Cyprus | MW138957 | Unpublished |
| *Aphanius fasciatus* | Italy | KJ709466 | [22] |
| *Aphanius fasciatus* | Italy | KJ709474 | [22] |
| *Aphanius fasciatus* | Tunisia | KJ552524 | [21] |
| *Aphanius fasciatus* | Tunisia | KJ552428 | [21] |
| *Aphanius fasciatus* | Italy | MH492717 | Unpublished |
| *Aphanius fasciatus* | Croatia | MH410032 | Unpublished |
| *Aphanius fasciatus* | Italy | MH492718 | Unpublished |
| *Anatolichthys danfordii* | Türkiye | KJ834531 | [23] |
| *Anatolichthys villwocki* | Türkiye | KJ834551 | [23] |
| *Anatolichthys maeandricus* | Türkiye | KJ552543 | [21] |
| *Anatolichthys transgrediens* | Türkiye | KJ552452 | [21] |
| *Aphaniops kruppi* | Oman | OP649683 | [24] |
| *Aphaniops hormuzensis* | Iran | MZ666383 | [25] |
| *Valencia hispanica* | Spain | OQ872784 | Unpublished |
| *Valencia robertae* | Greece | KF767514 | [26] |
| *Valencia robertae* | Greece | KF767519 | [26] |
| *Valencia letourneuxil* | Greece | KC106687 | [27] |

**Table 2.** Morphometric data of *Aphanius almiriensis* populations in Tuzla rheocrene spring area (Çanakkale, Türkiye).

| **Metric Characteristics** | **n: 17 ♀** | | | | **n: 10 ♂** | | | |
| --- | --- | --- | --- | --- | --- | --- | --- | --- |
|  | **mean** | **min** | **max** | **± SD** | **mean** | **min** | **max** | **± SD** |
| Standard length (mm) | 29.6 | 25.7 | 40.4 | 3.9 | 23.8 | 19.7 | 28.4 | 2.8 |
| ***In percent of standard length*** |  |  |  |  |  |  |  |  |
| Head length | 30.7 | 29.2 | 31.9 | 0.8 | 29.6 | 28.2 | 32.8 | 1.4 |
| Body depth at dorsal-fin origin | 22.9 | 21.0 | 24.4 | 1.1 | 22.0 | 20.1 | 24.9 | 1.6 |
| Body width at dorsal-fin origin | 10.8 | 8.4 | 13.7 | 1.5 | 9.5 | 7.2 | 13.4 | 1.8 |
| Predorsal length | 65.2 | 63.7 | 66.9 | 0.9 | 63.8 | 62.0 | 68.1 | 2.0 |
| Postdorsal length | 24.8 | 22.2 | 27.1 | 1.4 | 27.2 | 24.2 | 30.0 | 1.7 |
| Prepelvic length | 50.8 | 49.5 | 52.5 | 0.9 | 49.6 | 48.0 | 53.1 | 1.5 |
| Preanal length | 68.9 | 65.6 | 71.3 | 1.5 | 65.3 | 64.3 | 67.5 | 1.0 |
| Distance between pectoral and pelvic fin origin | 22.6 | 21.4 | 25.1 | 0.9 | 20.2 | 18.5 | 24.6 | 1.7 |
| Distance between pelvic and anal fin origin | 19.0 | 17.1 | 20.8 | 1.1 | 16.8 | 15.9 | 18.2 | 0.8 |
| Depth of caudal peduncle | 14.4 | 12.8 | 15.9 | 0.7 | 14.9 | 13.5 | 17.2 | 1.1 |
| Length of caudal peduncle | 21.7 | 19.4 | 23.9 | 1.4 | 24.0 | 22.0 | 25.9 | 1.2 |
| Pectoral fin length | 19.2 | 17.2 | 20.8 | 1.0 | 20.8 | 18.9 | 22.5 | 1.1 |
| Pelvic fin length | 10.9 | 9.7 | 11.8 | 0.6 | 10.7 | 9.2 | 12.1 | 1.1 |
| ***In percent of head length*** |  |  |  |  |  |  |  |  |
| Head depth at posterior of preoperculum | 64.3 | 59.7 | 72.5 | 2.9 | 65.7 | 63.0 | 69.0 | 2.1 |
| Head depth at eye | 53.2 | 49.8 | 58.8 | 2.6 | 54.6 | 49.6 | 59.9 | 3.3 |
| Snout length | 31.7 | 29.2 | 34.3 | 1.5 | 27.9 | 26.1 | 30.0 | 1.2 |
| Eye diameter | 31.0 | 24.5 | 34.0 | 2.6 | 31.3 | 29.3 | 33.8 | 1.6 |
| Postorbital distance | 45.4 | 43.5 | 48.1 | 1.3 | 45.6 | 43.7 | 47.6 | 1.4 |
| Maximum head width | 70.2 | 66.1 | 80.3 | 3.3 | 69.5 | 65,8 | 75.5 | 3.5 |
| Interorbital width | 39.0 | 36.5 | 45.0 | 2.1 | 37.3 | 34.3 | 41.0 | 2.5 |

**Table 3.** Genetic p-distances based on mitochondrial COI region of the species used in this study (The record marked in underlined was identified in this study, the other records were retrieved from GenBank).

|  | Species | 1 | 2 | 3 | 4 | 5 | 6 | 7 | 8 | 9 | 10 | 11 | 12 | 13 | 14 | 15 | 16 | 17 | 18 | 19 | 20 | 21 | 22 | 23 | 24 |
| --- | --- | --- | --- | --- | --- | --- | --- | --- | --- | --- | --- | --- | --- | --- | --- | --- | --- | --- | --- | --- | --- | --- | --- | --- | --- |
| 1 | *A. almiriensis* |  |  |  |  |  |  |  |  |  |  |  |  |  |  |  |  |  |  |  |  |  |  |  |  |
| 2 | *A. almiriensis* | 0.000 |  |  |  |  |  |  |  |  |  |  |  |  |  |  |  |  |  |  |  |  |  |  |  |
| 3 | *A. almiriensis* | 0.000 | 0.000 |  |  |  |  |  |  |  |  |  |  |  |  |  |  |  |  |  |  |  |  |  |  |
| 4 | *A. fasciatus* | 0.031 | 0.031 | 0.031 |  |  |  |  |  |  |  |  |  |  |  |  |  |  |  |  |  |  |  |  |  |
| 5 | *A. fasciatus* | 0.034 | 0.034 | 0.034 | 0.003 |  |  |  |  |  |  |  |  |  |  |  |  |  |  |  |  |  |  |  |  |
| 6 | *A. fasciatus* | 0.037 | 0.037 | 0.037 | 0.010 | 0.008 |  |  |  |  |  |  |  |  |  |  |  |  |  |  |  |  |  |  |  |
| 7 | *A. fasciatus* | 0.039 | 0.039 | 0.039 | 0.013 | 0.010 | 0.008 |  |  |  |  |  |  |  |  |  |  |  |  |  |  |  |  |  |  |
| 8 | *A. fasciatus* | 0.031 | 0.031 | 0.031 | 0.000 | 0.003 | 0.010 | 0.013 |  |  |  |  |  |  |  |  |  |  |  |  |  |  |  |  |  |
| 9 | *A. danfordii* | 0.102 | 0.102 | 0.102 | 0.112 | 0.110 | 0.107 | 0.112 | 0.112 |  |  |  |  |  |  |  |  |  |  |  |  |  |  |  |  |
| 10 | *A. maeandricus* | 0.120 | 0.120 | 0.120 | 0.120 | 0.117 | 0.125 | 0.123 | 0.120 | 0.097 |  |  |  |  |  |  |  |  |  |  |  |  |  |  |  |
| 11 | *A. transgrediens* | 0.117 | 0.117 | 0.117 | 0.131 | 0.128 | 0.125 | 0.123 | 0.131 | 0.070 | 0.068 |  |  |  |  |  |  |  |  |  |  |  |  |  |  |
| 12 | *A. villwocki* | 0.131 | 0.131 | 0.131 | 0.131 | 0.128 | 0.125 | 0.123 | 0.131 | 0.091 | 0.086 | 0.094 |  |  |  |  |  |  |  |  |  |  |  |  |  |
| 13 | *A. kruppi* | 0.183 | 0.183 | 0.183 | 0.175 | 0.178 | 0.175 | 0.180 | 0.175 | 0.191 | 0.198 | 0.191 | 0.196 |  |  |  |  |  |  |  |  |  |  |  |  |
| 14 | *A. hormuzensis* | 0.188 | 0.188 | 0.188 | 0.180 | 0.183 | 0.180 | 0.185 | 0.180 | 0.188 | 0.185 | 0.185 | 0.185 | 0.026 |  |  |  |  |  |  |  |  |  |  |  |
| 15 | *A. almiriensis* | 0.003 | 0.003 | 0.003 | 0.034 | 0.037 | 0.039 | 0.042 | 0.034 | 0.104 | 0.117 | 0.115 | 0.128 | 0.185 | 0.185 |  |  |  |  |  |  |  |  |  |  |
| 16 | *A. fasciatus* | 0.039 | 0.039 | 0.039 | 0.013 | 0.010 | 0.008 | 0.000 | 0.013 | 0.112 | 0.123 | 0.123 | 0.123 | 0.180 | 0.185 | 0.042 |  |  |  |  |  |  |  |  |  |
| 17 | *A. fasciatus* | 0.039 | 0.039 | 0.039 | 0.013 | 0.010 | 0.008 | 0.000 | 0.013 | 0.112 | 0.123 | 0.123 | 0.123 | 0.180 | 0.185 | 0.042 | 0.000 |  |  |  |  |  |  |  |  |
| 18 | *A. almiriensis* | 0.000 | 0.000 | 0.000 | 0.031 | 0.034 | 0.037 | 0.039 | 0.031 | 0.102 | 0.120 | 0.117 | 0.131 | 0.183 | 0.188 | 0.003 | 0.039 | 0.039 |  |  |  |  |  |  |  |
| 19 | *A. almiriensis* | 0.000 | 0.000 | 0.000 | 0.031 | 0.034 | 0.037 | 0.039 | 0.031 | 0.102 | 0.120 | 0.117 | 0.131 | 0.183 | 0.188 | 0.003 | 0.039 | 0.039 | 0.000 |  |  |  |  |  |  |
| 20 | *A. fasciatus* | 0.037 | 0.037 | 0.037 | 0.010 | 0.008 | 0.000 | 0.008 | 0.010 | 0.107 | 0.125 | 0.125 | 0.125 | 0.175 | 0.180 | 0.039 | 0.008 | 0.008 | 0.037 | 0.037 |  |  |  |  |  |
| 21 | *V. robertae* | 0.214 | 0.214 | 0.214 | 0.222 | 0.219 | 0.217 | 0.219 | 0.222 | 0.196 | 0.217 | 0.214 | 0.217 | 0.235 | 0.232 | 0.217 | 0.219 | 0.219 | 0.214 | 0.214 | 0.217 |  |  |  |  |
| 22 | *V. robertae* | 0.214 | 0.214 | 0.214 | 0.222 | 0.219 | 0.217 | 0.219 | 0.222 | 0.196 | 0.217 | 0.214 | 0.217 | 0.235 | 0.232 | 0.217 | 0.219 | 0.219 | 0.214 | 0.214 | 0.217 | 0.000 |  |  |  |
| 23 | *V. letourneuxi* | 0.214 | 0.214 | 0.214 | 0.222 | 0.219 | 0.217 | 0.219 | 0.222 | 0.198 | 0.219 | 0.217 | 0.219 | 0.235 | 0.232 | 0.217 | 0.219 | 0.219 | 0.214 | 0.214 | 0.217 | 0.003 | 0.003 |  |  |
| 24 | *V.hispanica* | 0.230 | 0.230 | 0.230 | 0.217 | 0.214 | 0.214 | 0.214 | 0.217 | 0.201 | 0.196 | 0.204 | 0.188 | 0.201 | 0.191 | 0.227 | 0.214 | 0.214 | 0.230 | 0.230 | 0.214 | 0.164 | 0.164 | 0.167 |  |

**Table 4.** Physico-chemical parameters measured in the study area (Çanakkale, Türkiye).

| **Stations** | **Seasons** | **WT (°C)** | **pH** | **Salinity (ppt)** | **EC (µS/cm)** | **DO (mg/L)** |
| --- | --- | --- | --- | --- | --- | --- |
| Site 1 | Winter | 20.2 | 7.48 | 61.14 | 77.82 | 9.41 |
|  | Spring | 28.2 | 6.95 | 61.09 | 88.19 | 7.38 |
|  | Summer | 31.5 | 6.55 | 60.58 | 87.80 | 6.20 |
|  | Autumn | 27.3 | 6.82 | 59.21 | 80.34 | 8.92 |
| Site 2 | Winter | 23.7 | 6.99 | 60.7 | 76.3 | 8.94 |
|  | Spring | 37.1 | 6.95 | 59.8 | 79.9 | 7.19 |
|  | Summer | 42.7 | 6.62 | 55.2 | 75.0 | 5.51 |
|  | Autumn | 40.0 | 6.81 | 47.7 | 63.9 | 7.49 |

**Figure 1.**

**Figure 2.**

**Figure 3.**

**Figure 4**

**Figure 5**

**Figure 6**

**Figure 7**

**Figure 8.**
