# Supplementary material for: Force-dependent development of the myodural bridge in rats: The impact of Integrin α7
Source: PLoS One. 2025 Aug 4;20(8):e0329754. doi: 10.1371/journal.pone.0329754 (PMC12321098; doi:10.1371/journal.pone.0329754)
Supplement: Attachment — Submitted filename: Response to Reviewers.docx [file pone.0329754.s012.docx]

Dear Editors and Reviewers,

Thank you for your meticulous review and valuable suggestions on our manuscript! We have carefully considered your comments, and the responses to the revision suggestions are as follows:

1. The macroscopic evidence of MDB dissection is provided in the Supplementary Materials. The evidence of compliance with the ARRIVE guidelines is reflected in the revised manuscript.
2. Regarding the issue of transcriptome data: First and foremost, we would like to express our sincere gratitude to the Editors and Reviewers for the precious advice on our manuscript! We sincerely apologize for any inadequacies in our initial handling of the transcriptome data analysis. Our team has thoroughly discussed the comment of “adding more content related to transcriptome data”. After careful consideration in combination with the subsequent research plan, we have decided, following repeated and prudent deliberation, to remove this part of the content from this article. We sincerely hope for your understanding and support. The explanations are as follows:

(1) The transcriptome data are not the core content of this study, and their removal does not affect the integrity of this article:

The core objective of this study is to explore the role of the target gene ITGA7 in the development process of MDBC, and to elucidate the force - dependent developmental mechanism of the myodural bridge (MDB). In the preliminary exploration stage, through literature research, it was found that the Integrins, particularly ITGA7, are critical for skeletal muscle attachment to connective tissues, and their function is involved in the transmission of lateral and longitudinal forces in skeletal muscle. Moreover, the development of MDB itself is inseparable from force transmission. Given the force - related characteristics of ITGA7 and the dependence of MDB development on force transmission, we initially speculated that ITGA7 might be involved in MDB development. Subsequently, we carried out the mRNA expressions of itga7 at the key developmental stages of MDBC in rats. The results showed that ITGA7 exhibited significant differential expression at different development stages. Even after excluding the transcriptome-related content, the rationale for this study on ITGA7 can still be elucidated. The above statement can fully explain the starting point of this research without relying on the detailed data from transcriptome sequencing in mice.

(2) Research on transcriptomics is ongoing, and detailed results will be published soon:

Currently, our research team is performing in-depth analyses and mining of transcriptome data related to MDB development, alongside experimental validation. Subsequently, we will also upload the complete data to the GEO database. Therefore, elaborating on this in the current article would lead to redundant content and deviate from the current research focus.

(3) Specific adjustment plan for removing the transcriptome content:

To ensure the logical coherence of the article, we will make the following modifications:

Introduction section: Delete the original paragraph related to the transcriptome.

Materials and Methods section: Delete the method for transcriptome sequencing.

Results section: The subtitle “Transcriptome sequencing reveals the potential value of ITGA7 in the developmental regulation of MDBC” is deleted.

Discussion section: Add explanations regarding the limitations of the transcriptomic data, for example:

“A more comprehensive and in - depth exploration of the transcriptomic data related to MDB development is still lacking. Therefore, in future studies, in - depth mining of such data will be crucial for identifying the regulation genes and signaling pathways, and ultimately providing a more complete understanding of the molecular mechanisms underlying MDB development.”

Thank you again for your professional advice! If you think that some transcriptome information still needs to be retained, we can provide the relevant content in the Supplementary Materials for readers' reference.

Thank you again for your professional comments! If there are any other suggestions, we will fully cooperate with the revisions.

Best regards,

Wei Ma

Hong-jin Sui

**Reviewer #1:**

**Introduction：**

1. **The content regarding CSF circulation has been removed, and repetitive descriptions of MDB composition have been revised.**

We have carefully reviewed the text and reorganized the paragraphs to ensure a more coherent progression of ideas. Specifically, we have eliminated the redundant descriptions of cerebrospinal fluid (CSF) circulation, removing overlapping statements about its role in maintaining CSF homeostasis. Similarly, we have streamlined the descriptions of the MDB's composition. Instead of separately stating that “the human MDB is a tendinous-like structure with parallel arranged collagen type I fibers” and later repeating that “the MDB, like tendons, is primarily composed of strong collagen fibril arrays”, we have combined these points into a single, concise description. This approach not only enhances readability but also makes the text more efficient in conveying key information about the MDB. We believe these changes have significantly improved the quality of the Introduction, and we appreciate your guidance in helping us refine our manuscript.

2. **Revised Logical Flow: From General MDB Characterization to Specific Structural Details：**

We reorganized the content in a hierarchical manner. First, we began with the basic anatomical definition of the MDB, detailing its location between suboccipital muscles and the spinal dura mater, its fiber origins, and its role in forming the MDBC. This provides a foundational understanding of the structure. Next, we presented the comparative anatomical evidence of the MDB's conservation across species, followed by its established physiological functions, highlighting its importance in various biological processes. Finally, we delved into the MDB's composition, specifically its collagen - based structure for force transmission, and then introduced its developmental process in rat embryos and human fetuses. This sequence ends with the speculation on the role of mechanical stress in MDB maturation and the need for further research, creating a clear flow from general anatomy and function to specific structural and developmental details. We believe this restructuring effectively improves the logical clarity of the text.

### **MM：1. Power Analysis for Sample Size Justification**

In the "Materials and Methods(Sample Size Determination)" section, we have included a power analysis to justify the sample sizes used. We employed G*Power 3.1.9.7 for this purpose. Under the "t tests" family, we selected the "Correlation: Point biserial model" and adopted an a priori approach.

We set the significance level (α) at 0.05, aiming for a 5% probability of a Type I error. The desired statistical power (1 - β) was set at 0.8, meaning we strived for an 80% chance of detecting a true effect. Based on relevant prior studies, we estimated the effect size to be 0.5. Given our lack of directional assumptions, a two - tailed test was chosen.

Upon inputting these parameters into the software and executing the calculation, the output revealed that a total sample size of 26 was required. The non - centrality parameter (δ) was 2.9439203, the critical t - value was 2.0638986, and the degrees of freedom (Df) were 24. The actual power calculated by the software was 0.8063175, which was close to our target power of 0.8. Considering potential animals dropout, we planned to use 32 mice per group to ensure sufficient statistical power. This addition provides a more robust scientific basis for our sample size determination.

### 2. **Addition of an Untreated Control Group**

To rule out non - specific effects from the injection procedure, we added a sham group. This group underwent the same procedures as the shRNA-ITGA7 group, except that it only received needle insertion without any injection. We conducted a morphological analysis on this sham group and compared the results with the shRNA - NC group. As expected, no significant differences were observed between the sham group and the shRNA-NC group. Given that the primary focus of our study is to investigate the specific role of ITGA7 in MDB formation through lentivirus-mediated gene knockdown, the subsequent experiments were mainly designed to explore the differences between the shRNA - NC group and shRNA - ITGA7 group, so we did not include this group in the subsequent experiments.

### **3. Quantitative Analyses of Picrosirius Red and Masson Staining Images**

Regarding the Picrosirius Red and Masson staining images, we have carried out quantitative analyses of collagen fiber density or optical density measurements. We used image J to analyze the images. In the "Materials and Methods" section, we have described the specific steps of the image analysis. The results of these quantitative analyses are presented in the "Results" section, which will enable a more objective and accurate assessment of the changes in collagen fiber content or distribution among different groups.（S4C and S5E Figs)

**R：Conduct a functional enrichment analysis using Gene Ontology (GO) or KEGG pathway to identify other relevant genes. This will strengthen the conclusions about molecular players in MDB development.**

We sincerely appreciate your insightful suggestion regarding the functional enrichment analysis. Given that we had to remove the transcriptomic data section from the current manuscript due to its limited relevance to the main research focus (as previously explained). This is exactly in line with our future research plan. As we mentioned in the limitations section, we are committed to conducting a detailed analysis of the MDB development - related transcriptomic data. In the follow-up studies, we will definitely perform GO and KEGG pathway enrichment analyses to systematically identify other genes associated with MDB development, which will strengthen our understanding of the molecular mechanisms involved. This comprehensive analysis will be a crucial part of our future research.

**Discussion:**

1. **Discuss the relevance of these additional genes potential limitations, such as extrapolating findings from rats to humans and possible interactions with other extracellular matrix proteins that might influence MDB development.**

We have added a dedicated paragraph on limitations in the Discussion section, which covers: (1) considerations for translating findings from rodent to human anatomical structures; (2) potential interactions with other extracellular matrix components; (3) the need for a comprehensive exploration of transcriptomic data to fully elucidate the molecular mechanisms of MDB development. These additions provide a more comprehensive and objective perspective while maintaining the focus on our core finding regarding the role of ITGA7 in the development of the myodural bridge (MDB).

2. **Provide a more detailed discussion on the potential clinical implications of the findings, including the role of the MDB in CSF circulation and its relationship to cervicogenic headaches.**

In the discussion section, we have supplemented a more detailed exploration of the potential clinical implications of the findings, particularly focusing on the role of the myodural bridge (MDB) in cerebrospinal fluid (CSF) circulation and its association with cervicogenic headaches. This study mainly aims to clarify the structural effects of ITGA7 knockdown on MDBC development. It is worth noting that in our ongoing follow-up studies, we will systematically explore the correlations between MDBC developmental abnormalities, CSF circulation, pain mechanisms, and their clinical significance. These studies will include behavioral pain assessments and evaluations of relevant indicators in animal models to further validate the clinical significance of these findings.

**Reviewer #2:**

1. **It is not sufficiently clear how the sample size for each group was calculated.**

We greatly appreciate your valuable suggestions. All experiments were performed at least in triplicate, and some were conducted with four times to clarify the results more effectively. For Masson staining and Sirius red staining, we used different sections from the same animals. The mRNA expression of ITGA7 at different developmental stages (E16, P0, P7, and P14) was analyzed in wild-type Sprague-Dawley (SD) rats, qRT-PCR were performed in triplicate, so the number of animals is 12. The Sham group included 4 animals to exclude potential confounding effects caused by needle insertion.

We have rechecked the number of cases for each experiment, and the specific quantities are shown in the following table:

| **Experimental Method** | **Number of Animals in shRNA - ITGA7 Group** | **Number of Animals in shRNA - NC Group** | **Notes** |
| --- | --- | --- | --- |
| **Initial Enrollment** | **38** | **32** | Pre-GFP screening |
| **Valid GFP+ Samples (Final)** | **35** | **29** | RCDmi coverage |
| qRT - PCR | 3 | 3 |  |
| Western blotting | shRNA - 1  shRNA - 2  shRNA-3, each 3 | 3 | Three samples for each of shRNA - 1 and shRNA - 2 were not included in the total sample size. |
| Histological sectioning and staining | shRNA - 1: 1  shRNA - 2: 1  shRNA-3: 1 | P7: 1 | In morphology, shRNA-1 and shRNA-2 used for screening effective targets were not included in the total sample size. |
| Histological sectioning and staining | P7: 3  P14: 4 | P7: 3  P14: 4 | Masson and Sirius red staining |
| Immunostaining | 3 | 3 | Independent cohort |
| Uniaxial tensile test | 3 | 3 | Independent cohort |
| Scanning electron microscopy | 3×2（two time points） | 3×2（two time points） |  |
| Transmission microscopy | 3 | 3 | Independent cohort |
| **Total** | 35 | 29 |  |

In the "Materials and Methods(Sample Size Determination)" section, we have included a power analysis to justify the sample sizes used. We employed G*Power 3.1.9.7 for this purpose. Under the "t tests" family, we selected the "Correlation: Point biserial model" and adopted an a priori approach.

We set the significance level (α) at 0.05, aiming for a 5% probability of a Type I error. The desired statistical power (1 - β) was set at 0.8, meaning we strived for an 80% chance of detecting a true effect. Based on relevant prior studies, we estimated the effect size to be 0.5. Given our lack of directional assumptions, a two - tailed test was chosen.

Upon inputting these parameters into the software and executing the calculation, the output revealed that a total sample size of 26 was required. The non - centrality parameter (δ) was 2.9439203, the critical t - value was 2.0638986, and the degrees of freedom (Df) were 24. The actual power calculated by the software was 0.8063175, which was close to our target power of 0.8. Considering potential mouse dropout, we planned to use 32 mice per group to ensure sufficient statistical power. This addition provides a more robust scientific basis for our sample size determination.

In order to meet the requirements of the ARRIVE guidelines, the Abstract summarizes the research objectives, animal species, strains, and genders, key methods, main findings, and research conclusions. The Introduction includes the scientific background and explanations of the animal species and models used. The research questions and objectives are clearly described. Regarding the ethical statement, our study has obtained approval from the Animal Ethics Review Committee of Dalian Medical University (No: AEE21085). Interventions to reduce pain are included in the experimental procedures. The results are interpreted in relation to the research objectives and hypotheses, and the limitations are clarified. The manuscript contains data access statements and a declaration of interests.

**ARRIVE guidelines：**

| Study design  1 For each experiment, provide brief details of study design including:   a. The groups being compared, including control groups. If no control group has been used, the rationale should be stated.   b. The experimental unit (e.g. a single animal, litter, or cage of animals) | Materials and methods  Animal model and Housing  (Lines 141-155) |
| --- | --- |
| Sample size  2 a. Specify the exact number of experimental units allocated to each group, and the total number in each experiment. Also indicate the total number of animals used. b. Explain how the sample size was decided. Provide details of any a priori sample size calculation, if done. | Materials and methods  Sample Size Determination  (Lines 184-190) |
| Inclusion and exclusion criteria  3 a. Describe any criteria used for including and excluding animals (or experimental units) during the experiment, and data points during the analysis. Specify if these criteria were established a priori. If no criteria were set, state this explicitly.  b. For each experimental group, report any animals, experimental units or data points not included in the analysis and explain why. If there were no exclusions, state so. c. For each analysis, report the exact value of n in each experimental group. | Materials and methods  Sample Size Determination  Animal model and Housing  ((Lines 141-155)(Lines 184-190) |
| Randomisation  4 a. State whether randomisation was used to allocate experimental units to control and treatment groups. If done, provide the method used to generate the randomisation sequence.  b. Describe the strategy used to minimise potential confounders such as the order of treatments and measurements, or animal/cage location. If confounders were not controlled, state this explicitly | Materials and methods  Blinding and Outcome Measures  Animal model and Housing  (Lines 141-155)(Lines 178-182) |
| Blinding  5 Describe who was aware of the group allocation at the different stages of the experiment (during the allocation, the conduct of the experiment, the outcome assessment, and the data analysis). | Materials and methods  Blinding and Outcome Measures  (Lines 178-182) |
| Outcome measures  6 a. Clearly define all outcome measures assessed (e.g. cell death, molecular markers, or behavioural changes).  b. For hypothesis-testing studies, specify the primary outcome measure, i.e. the outcome measure that was used to determine the sample size | Materials and methods  (Lines 178-182) (Lines 184-190) |
| Statistical methods  7 a. Provide details of the statistical methods used for each analysis, including software used.  b. Describe any methods used to assess whether the data met the assumptions of the statistical approach, and what was done if the assumptions were not met. | Materials and methods  Statistical analysis  (Lines 285-290) |
| Experimental animals  8 a. Provide species-appropriate details of the animals used, including species, strain and substrain, sex, age or developmental stage, and, if relevant, weight. b. Provide further relevant information on the provenance of animals, health/immune status, genetic modification status, genotype, and any previous procedures | Materials and methods  Animal model and Housing  (Lines 141-155) |
| Experimental procedures  9 For each experimental group, including controls, describe the procedures in enough detail to allow others to replicate them, including: a. What was done, how it was done and what was used. b. When and how often. c. Where (including detail of any acclimatisation periods). d. Why (provide rationale for procedures) | Materials and methods  Animal model and Housing  Lentiviral Vector Preparation and Injection  (Lines 140-155) (Lines 157-170) |
| Results  10 For each experiment conducted, including independent replications, report:  a. Summary/descriptive statistics for each experimental group, with a measure of variability where applicable (e.g. mean and SD, or median and range).  b. If applicable, the effect size with a confidence interval. | Results  (Lines 292-628)  Materials and methods  Statistical analysis  (Lines 285-290) |

| Abstract  11 Provide an accurate summary of the research objectives, animal species, strain and sex,  key methods, principal findings, and study conclusions | Abstract  (Lines 17-47) |
| --- | --- |
| Background  12 a. Include sufficient scientific background to understand the rationale and context for the study, and explain the experimental approach. b. Explain how the animal species and model used address the scientific objectives and, where appropriate, the relevance to human biology. | Introduction  (Lines 49-129) |
| Objectives  13 Clearly describe the research question, research objectives and, where appropriate, specific hypotheses being tested. | Introduction  (Lines 111-129) |
| Ethical statement  14 Provide the name of the ethical review committee or equivalent that has approved the use of animals in this study, and any relevant licence or protocol numbers (if applicable). If ethical approval was not sought or granted, provide a justification. | Materials and methods  Ethical Statements  (Lines 132-139) |
| Housing and husbandry  15 Provide details of housing and husbandry conditions, including any environmental enrichment | Materials and methods  Animal model and Housing  （Lines 141-155） |
| Animal care and monitoring  16 a. Describe any interventions or steps taken in the experimental protocols to reduce pain, suffering and distress.   b. Report any expected or unexpected adverse events. c. Describe the humane endpoints established for the study, the signs that were monitored and the frequency of monitoring. If the study did not have humane endpoints, state this | Materials and methods  Animal model and Housing  (Lines 141-155) |
| Interpretation/scientific implications  17 a. Interpret the results, taking into account the study objectives and hypotheses, current theory and other relevant studies in the literature.  b. Comment on the study limitations including potential sources of bias, limitations of the animal model, and imprecision associated with the results. | Results  Discussion  (Lines 292-628) (Lines 735-748) |
| Generalisability/ translation  18 Comment on whether, and how, the findings of this study are likely to generalise to other species or experimental conditions, including any relevance to human biology (where appropriate). | Discussion  (Lines 736-739) |
| Protocol registration  19 Provide a statement indicating whether a protocol (including the research question, key design features, and analysis plan) was prepared before the study, and if and where this protocol was registered. | Data availability  (Lines 774-776） |
| Data access  20 Provide a statement describing if and where study data are available | Data availability  (Lines 774-776） |
| Declaration of interests  21 a. Declare any potential conflicts of interest, including financial and non-financial. If none exist, this should be stated.  b. List all funding sources (including grant identifier) and the role of the funder(s) in the design, analysis and reporting of the study. | Declaration of interests  (Lines 778-782) |

2. **Additionally, as a minor suggestion, the sentence "Future research should investigate the clinical implications of abnormal MDBC development, particularly in relation to headaches." should be removed from the conclusions section. The authors might consider relocating it to the Discussion section, as it is not directly related to the findings of the current study.**

We sincerely appreciate your meticulous review and the valuable suggestion. In response to your recommendation, we have promptly removed the sentence "Future research should investigate the clinical implications of abnormal MDBC development, particularly in relation to headaches." from the Conclusions section. As you pointed out, this statement indeed diverges from the direct findings of our current study. We have carefully relocated it to the Discussion section, where it now fits more appropriately. This adjustment helps to maintain the clarity and coherence of the Conclusions section, ensuring that it focuses solely on summarizing our current research results.

**Reviewer #3:**

**Abstract:**

**1. Clarification on Multiple MDBs and Fibrous Connections:**

We acknowledge your point that the number of MDBs varies by species, and that fibrous connections exist not only between the duramater and suboccipital muscles but also with suboccipital ligaments. In previous studies, we regarded MDB as a whole. Hack et al. (1995) proposed the concept of the myodural bridge (MDB)describing the MDB as a dense fibrous tissue bridge that functionally and anatomically connects the rectus capitis posterior minor muscle to the spinal dura mater (SDM), while passing through the posterior atlanto‐occipital interspace (PAOI). In the future, we will pay attention to the usage of "MDB" to ensure it is not misinterpreted as referring to "only one“.

**2. Revision of "Cerebrospinal Fluid Dynamics":**

We have made the revisions as per your instructions. The manuscript has been adjusted to clarify this distinction.

**3. Removal of Unsupported Statement in Abstract:**

The last sentence of the Abstract, "... and its contribution to cerebrospinal fluid circulation dynamics," has been deleted.

**Introduction:**

**1.It should not focus on the circulation of the cerebrospinal fluid but on the MDB and/or MDBC**

We have eliminated the redundant descriptions of cerebrospinal fluid (CSF) circulation, removing overlapping statements about its role in maintaining CSF homeostasis.

**Results:** **the dissection and macroscopic appearance of the regions studied and their histology.**

We can provide relevant images, including the exposure process of the rectus capitis posterior major muscle according to anatomical layers:

Incision of the skin → Dissection of subcutaneous tissue → Remove the superfical muscles and exposure the RCPma → Identification of the atlantooccipital space → Harvesting of the rectus capitis posterior major and rectus capitis posterior minor muscles together with the dura mater.(S1 Fig)

**In fetuses and embryos that are also small, in which the size of the MDBs is extremely small, it is important that the experiments have been carried out on the appropriate tissues and not on other nearby ones.**

For the isolation of embryonic MDBC, we performed a standardized microdissection protocol under stereomicroscopic guidance. The dissection procedure was conducted as follows:

Tissue Preparation: Embryonic specimens were first sagittally bisected along the midline to expose the atlanto-occipital region.

Landmark Identification: The occipital bone and atlas (C1 vertebra) were identified as key anatomical references.

Targeted Dissection: The entire tissue within the atlanto-occipital space was carefully excised to ensure complete MDBC retrieval (representative dissection images are provided in Supplementary Materials to illustrate the molecular experimental sampling).(S1 Fig)
