# Supplementary material for: Force-dependent development of the myodural bridge in rats: The impact of Integrin α7
Source: PLoS One. 2025 Aug 4;20(8):e0329754. doi: 10.1371/journal.pone.0329754 (PMC12321098; doi:10.1371/journal.pone.0329754)
Supplement: Attachment — Submitted filename: Response_to_Reviewers_auresp_2.docx [file pone.0329754.s013.docx]

Reviewer #1:

We sincerely appreciate your constructive comments, which have greatly enhanced the quality of our manuscript. All suggestions have been carefully addressed as follows:

1. Correct minor typographical errors, such as "invlove" → "involve."

All minor typographical errors, including "invlove" corrected to "involve," have been identified and revised in the manuscript.

2. Introduction: Residual focus on CSF dynamics, though reduced, could distract from the central mechanobiology hypothesis. Ensure all speculative statements regarding CSF dynamics and clinical analogies are clearly presented as hypotheses for future research rather than conclusions.

In the Introduction, we have rephrased all statements related to CSF dynamics and clinical analogies to explicitly frame them as hypotheses for future research, rather than conclusions, to avoid distracting from the central mechanobiology focus.

①“Notably, the upper cervical region harharbors a specialized biomechanical microenvironment critical for localized CSF regulation— the myodural bridge (MDB)[8-11], et al.”→“Notably, the upper cervical region harharbors a specialized biomechanical microenvironment that may be involved in localized CSF regulation— the myodural bridge (MDB)[8-11], ”

3. Consider adding in Results a summary table consolidating main quantitative results (e.g., collagen volume fraction, muscle force, gene expression) to aid reader comprehension.

A summary table has been added in the Results section.(S3 Table)

4. Discussion: Discussion on CSF dynamics and clinical analogies (e.g., Chiari malformations) should be presented as speculative, not definitive conclusions. Potential sex-based differences (given mixed-sex animals) are not addressed.

In the Discussion, we have adjusted the content on CSF dynamics and clinical analogies (e.g., Chiari malformations) to emphasize their speculative nature. Additionally, In the limitations of the discussion, we clarify that our mixed-sex animal model cannot analyze potential sex-based differences in the role of ITGA7 in MDB development.

①"This study provides further evidence that suboccipital muscle force is transmitted to the SDM via the MDB, acting as a dynamic driver of CSF circulation."→"This study suggests that suboccipital muscle force transmitted to the SDM via the MDB may act as a potential dynamic driver of CSF circulation."

②"The potential impact of this condition may be similar to that of cerebellar tonsillar herniation, known to disrupt CSF circulation."→"It is plausible that this condition could have effects analogous to cerebellar tonsillar herniation (a disorder associated with CSF circulation disruption), though such a link remains speculative and requires direct investigation."

③"Numerous studies have demonstrated that the MDB couples suboccipital muscle movements to cerebrospinal fluid (CSF) dynamics in human[11, 44] and animal models..."→"Numerous studies suggest that the MDB may couple suboccipital muscle movements to cerebrospinal fluid (CSF) dynamics in humans[11, 44] and animal models...".

④"These findings provide direct evidence for the role of the MDBC in headache... This preserves mechanical buffering, maintains CSF circulation, and reduces risks of headache and CSF leakage from CSF dynamic disorders[60]."→"These findings suggest a potential role of the MDBC in headache... This may help preserve mechanical buffering, support CSF circulation, and reduce potential risks of headache and CSF leakage related to CSF dynamic disorders[60], though clinical confirmation is needed."

Reviewer #2:

Thank you very much for your positive comments and valuable feedback on our manuscript. We truly appreciate your time and efforts in reviewing our work.

Reviewer #3:

We sincerely appreciate your valuable insight regarding the proprioceptive innervation of myodural bridges (MDBs). This is indeed an important aspect, and thank you for highlighting it. Our research team is currently conducting studies related to this area, and relevant articles will be published subsequently. We truly value your expertise and hope to further explore this interesting topic in future research.

Thank you again for your valuable contributions. We hope these revisions address your concerns adequately. Please let us know if further adjustments are needed.

Best regards,

Wei Ma

Hong-jin Sui
